# Draft genome sequence of cadmium-tolerant *Pseudomonas aeruginosa* strain PR-5 from industrial contaminated water

**DOI:** 10.1128/mra.00872-25

**Published:** 2025-12-04

**Authors:** Prerna Chauhan, Akansha Garg, Charanjeet Kaur, Mushtaq Ali, Pankaj Kumar Arora, Shahnaz Perveen, Alok Srivastava

**Affiliations:** 1 Department of Plant Science, M.J.P. Rohilkhand University, Bareilly, Uttar Pradesh, India; 2 Department of Microbiology, M.J.P. Rohilkhand University, Bareilly, Uttar Pradesh, India; 3 Department of Botany, Central University of Kashmir, Kashmir, India; DOE Joint Genome Institute, Berkeley, California, USA

**Keywords:** *Pseudomonas aeruginosa*, cadmium, chemotaxis, biofilm

## Abstract

We report the draft genome of a cadmium-resistant *Pseudomonas aeruginosa* strain, PR5 (6.36 Mb, 66.5% GC, 5,934 genes). Genomic analysis revealed it contains genes for metal detoxification, transport, biofilm formation, and chemotaxis. The analysis reveals the strain’s robust adaptability and its promising role in cadmium bioremediation applications.

## ANNOUNCEMENT

Cadmium is a toxic, non-biodegradable heavy metal released from industrial processes, posing major environmental and health hazards. Mitigating cadmium pollution is crucial, and microbial bioremediation offers one of the most effective and sustainable solutions (1). Cadmium-tolerant *Pseudomonas aeruginosa* PR-5 was obtained from a polluted water sample collected on 15 April 2023 at B.L. Agro Industries, Bareilly, Uttar Pradesh (28.40141°N, 79.36781°E). The sample was aseptically collected and immediately processed. Culturing on nutrient agar with increasing CdCl_₂_ concentrations (10–3,000 ppm) revealed PR-5’s resistance up to 2,500 ppm (2, 3). To explore the potential genes involved in cadmium tolerance and bioremediation, whole-genome sequencing was performed (4, 5).

Genomic DNA was extracted from a single purified colony of PR5 grown in LB broth at 30°C for 18–24 h under shaking conditions. The harvested cells were processed for DNA extraction using the QIAGEN Genomic-tip 20/G kit and fragmented enzymatically into 200–300 base pair segments using NEBNext Ultra II FS DNA Library Prep Kit (6, 7). Fragmented DNA was end-repaired to generate blunt ends by exonuclease and polymerase treatments. A single “A” nucleotide was then added to the 3′ ends, and loop adapters were added before cleavage with the USER (Uracil-Specific Excision Reagent) enzyme and AMPure beads were used for purification (8, 9). Library amplification was performed using NEBNext Ultra II Q5 master mix with Illumina universal and *Pseudomonas*-specific octamer primers in six PCR cycles, followed by further purification and elution. After verifying DNA library quality and size distribution, sequencing was conducted on the Illumina platform using 151 bp paired-end reads, generating a total of 5.6 million reads (10). Raw reads were quality-checked using FastQC (v0.12.1), aggregated with MultiQC (v1.25), and trimmed with TrimGalore (v0.6.4) to remove adapters and low-quality bases. High-quality trimmed reads were assembled *de novo* using Unicycler (v0.4.8) (11–13). The draft genome assembly spans 6.36 Mbp with an average 66.5% GC content and 84,819 sequencing depth (Table 1). It includes 75 contigs, with 3 predicted as plasmids by MOB-Suite (v3.1.4). Assembly metrics include *N*_50_ of 267.5 kb and *L*_50_ of 8. CheckM (v1.2.3) analysis revealed 99.63% completeness, based on *Pseudomonas*-specific marker set (14). Functional annotation of the PR-5 genome using PGAP (v6.9) revealed 11 genes associated with heavy metal-detoxification, nine metal-transporter genes, and one gene for cadmium detoxification, revealing its genetic ability to survive in metal-contaminated environments (15). Other than metal-associated, 9 biofilm and 42 chemotaxis-related genes are also present in the PR-5 strain (https://doi.org/10.6084/m9.figshare.30363619). BLASTn analysis of PR-5 16S rRNA sequence against the NCBI 16S rRNA database revealed 99.71% similarity to *P. aeruginosa* DSM 50071 confirming PR-5 as *P. aeruginosa* (16, 17). The genome *P. aeruginosa* PR-5 harbors genes for cadmium detoxification, chemotaxis, and biofilm formation. Its high-quality genome provides a unique resource for understanding heavy metal adaptation and developing bioremediation strategies.

**TABLE 1:** Detailed information of draft genome

Parameter	Value or results
Genetic element	Contig and plasmid
Genome size (bp)	6,361,437
Total number of contigs	75
Number of predicted plasmids	3
Plasmid pPAu1 73	JBLRXR010000073.1 (Contig 73)
Plasmid pPAu2 74	JBLRXR010000074.1 (Contig 74)
Plasmid pPAu3 75	JBLRXR010000075.1 (Contig 75)
Number of total genes	5934
GC content (%)	66.5%
Number of rRNAs	6
Number of tRNAs	58
GenBank accession number	JBLRXR000000000
Sequence Read Archive accession number	SRR31822932

## Data Availability

The whole-genome assembly of *P. aeruginosa* PR-5 is available in NCBI GenBank (accession no. JBLRXR000000000), and raw sequencing reads have been deposited in the NCBI Sequence Read Archive (SRA) under accession number SRR31822932.
